# Supplementary material for: In-Situ Sludge Reduction in Membrane-Controlled Anoxic-Oxic-Anoxic Bioreactor: Performance and Mechanism
Source: Membranes (Basel). 2022 Jun 27;12(7):659. doi: 10.3390/membranes12070659 (PMC9321052; doi:10.3390/membranes12070659)
Supplement: Supplementary file 1 [file membranes-12-00659-s001.zip › membranes-1777271-supplementary.pdf]

Supplementary Information

In-situ sludge reduction in membrane-controlled anoxic-oxic-anoxic bioreactor: Performance and mechanism

Chengyue Li [a,b,c,1], Tahir Maqbool [a,b,c,1] , Hongyu Kang [a,b,c] and Zhenghua Zhang [a,b,c*]

[a]Institute of Environmental Engineering & Nano-Technology, Tsinghua Shenzhen International Graduate School, Tsinghua University, Shenzhen 518055, Guangdong, China

[b]Guangdong Provincial Engineering Research Centre for Urban Water Recycling and Environmental Safety, Tsinghua Shenzhen International Graduate School, Tsinghua University, Shenzhen, 518055, Guangdong, China

[c]School of Environment, Tsinghua University, Beijing 100084, China

Membranes

Submitted on June 2022

[1]Co-first authors

*Corresponding author: zhenghua.zhang@sz.tsinghua.edu.cn (Z. Zhang)

Table S1. Chemical composition of synthetic wastewater.

Components	Concentration (mg/L)
Glucose	1000
NH_4Cl	140
KH_2PO_4	25
$NaNO_3$	40
$MgSO_4.7H_2O$	5
$FeCl_3$	2.33
$NaHCO_3$	Appropriate to maintain pH = 7.0

Table S2. The average ± standard deviation of basic quality parameters of effluent in three different bioreactors (n=25).

Items	Influent (mg/L)	Effluent (mg/L)			Removal (%)		
		$MBR_{control}$	AOA_{90}	AOA_{80}	$MBR_{control}$	AOA_{90}	AOA_{80}
**DOC**	399.44±2.36	2.32±0.81	3.38±1.21	3.14±1.71	>99.00	>99.00	>99.00
**TP**	6.08±0.17	0.07±0.14	0.62±0.46	0.65±0.25	98.72±2.25	89.82±7.51	89.28±4.16
**TN**	40.05±0.35	2.43±1.76	2.19±1.23	1.77±1.55	93.93±4.40	94.52±3.08	95.58±3.87

Table S3. The average of NH_4^+ and NO_3^- in the supernatant of different treatment units of MBRs (n=14).

Items	$MBR_{control}$			AOA_{90}			AOA_{80}		
	**Anoxic**	**Oxic**	**M-tank**	**Anoxic**	**Oxic**	**M-tank**	**Anoxic**	**Oxic**	**M-tank**
**NH_4^+ (mg/L)**	1.73±1.52	0.24±0.36	0.14±0.23	6.71±2.18	0.47±0.63	0.93±1.33	3.23±2.87	0.25±0.31	1.12±1.36
**NO_3^- (mg/L)**	0.18±0.32	1.62±1.43	1.60±1.57	0.14±0.16	1.05±0.77	0.20±0.61	0.21±0.21	1.57±1.08	0.62±0.75

Table S4. Sequencing of bacterial 16S rRNA gene along with alpha diversity of microbial taxa in three bioreactors.

Sample description	Sequencing results		Species richness	Species diversity	
	Effective tags	OTUs	Chao-1	Shannon	Simpson
$MBR_{control}$-A-0d	49148	1700	1700.3	6.78	0.033
$MBR_{control}$-O-0d	50916	1664	1664.5	6.65	0.0525
$MBR_{control}$-M-0d	50945	1652	1652.3	6.16	0.0837
AOA_{90}-A-0d	49437	1378	1378.4	6.38	0.0428
AOA_{90}-O-0d	53188	1285	1285.5	6.32	0.0428
AOA_{90}-M-0d	51434	1260	1260.5	5.82	0.0811
AOA_{80}-A-0d	47001	1803	1803.3	7.22	0.0289
AOA_{80}-O-0d	48234	1733	1733.3	7.3	0.0191
AOA_{80}-M-0d	45035	1745	1745.2	7.25	0.0246
$MBR_{control}$-A-45d	67700	1068	1068.5	6.18	0.072
$MBR_{control}$-O-45d	58027	984	984.6	5.78	0.102
$MBR_{control}$-M-45d	51426	1202	1202.2	5.56	0.133
AOA_{90}-A-45d	76204	1099	1099.2	6.67	0.0456
AOA_{90}-O-45d	67258	1155	1155.5	6.71	0.0439
AOA_{90}-M-45d	57062	1463	1463.1	7.13	0.0246
AOA_{80}-A-45d	65916	884	884.3	6.04	0.0639
AOA_{80}-O-45d	56788	1212	1212.1	5.83	0.0838
AOA_{80}-M-45d	56793	1115	1115.2	5.75	0.0695
$MBR_{control}$-A-90d	62360	988	988.9	5.26	0.129
$MBR_{control}$-O-90d	63004	959	959.7	6.14	0.0477
$MBR_{control}$-M-90d	47573	1088	1088.3	5.99	0.0606
AOA_{90}-A-90d	51723	952	953	5.74	0.0745
AOA_{90}-O-90d	66099	885	886.1	5.38	0.0602
AOA_{90}-M-90d	46637	1063	1063.2	5.78	0.0672
AOA_{80}-A-90d	54424	945	945.9	5.17	0.0961
AOA_{80}-O-90d	58247	971	972	5.3	0.117
AOA_{80}-M-90d	59330	756	756.7	3.91	0.213

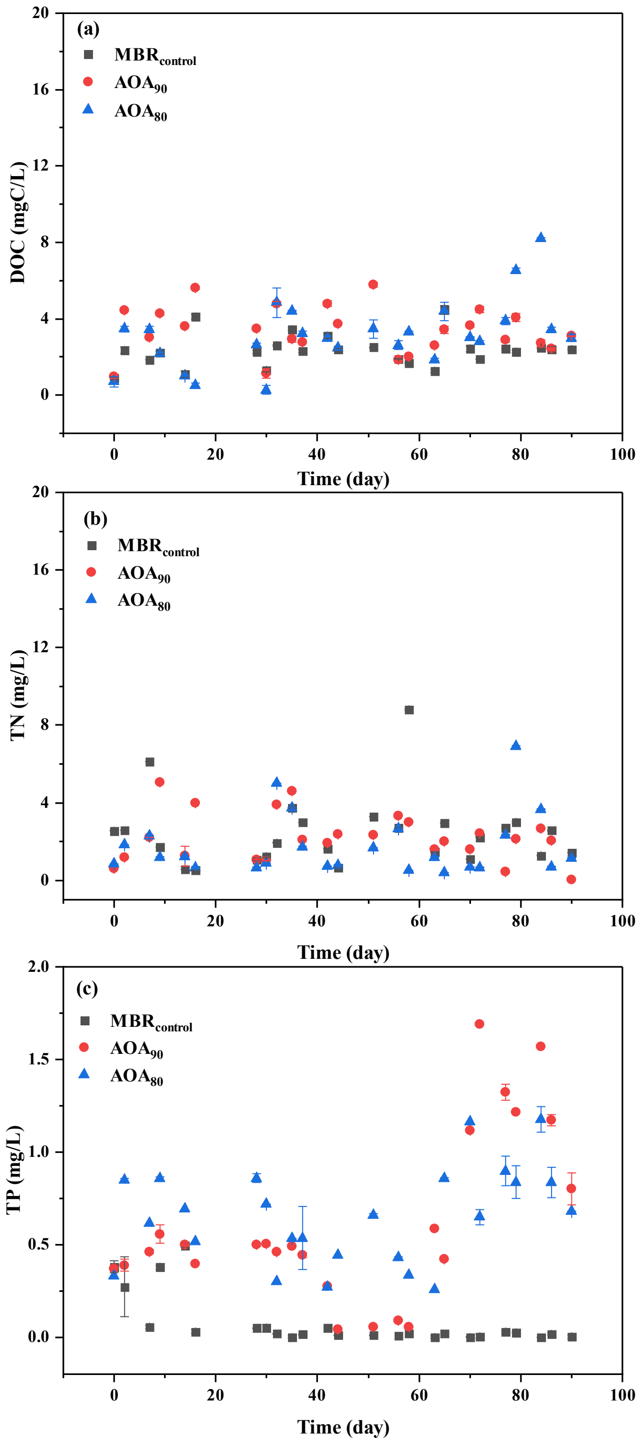

Figure S1. The temporal variations in basic quality parameters (DOC, TN, and TP) in effluents from MBRs ($MBR_{control}$, AOA_{90}, and AOA_{80}).

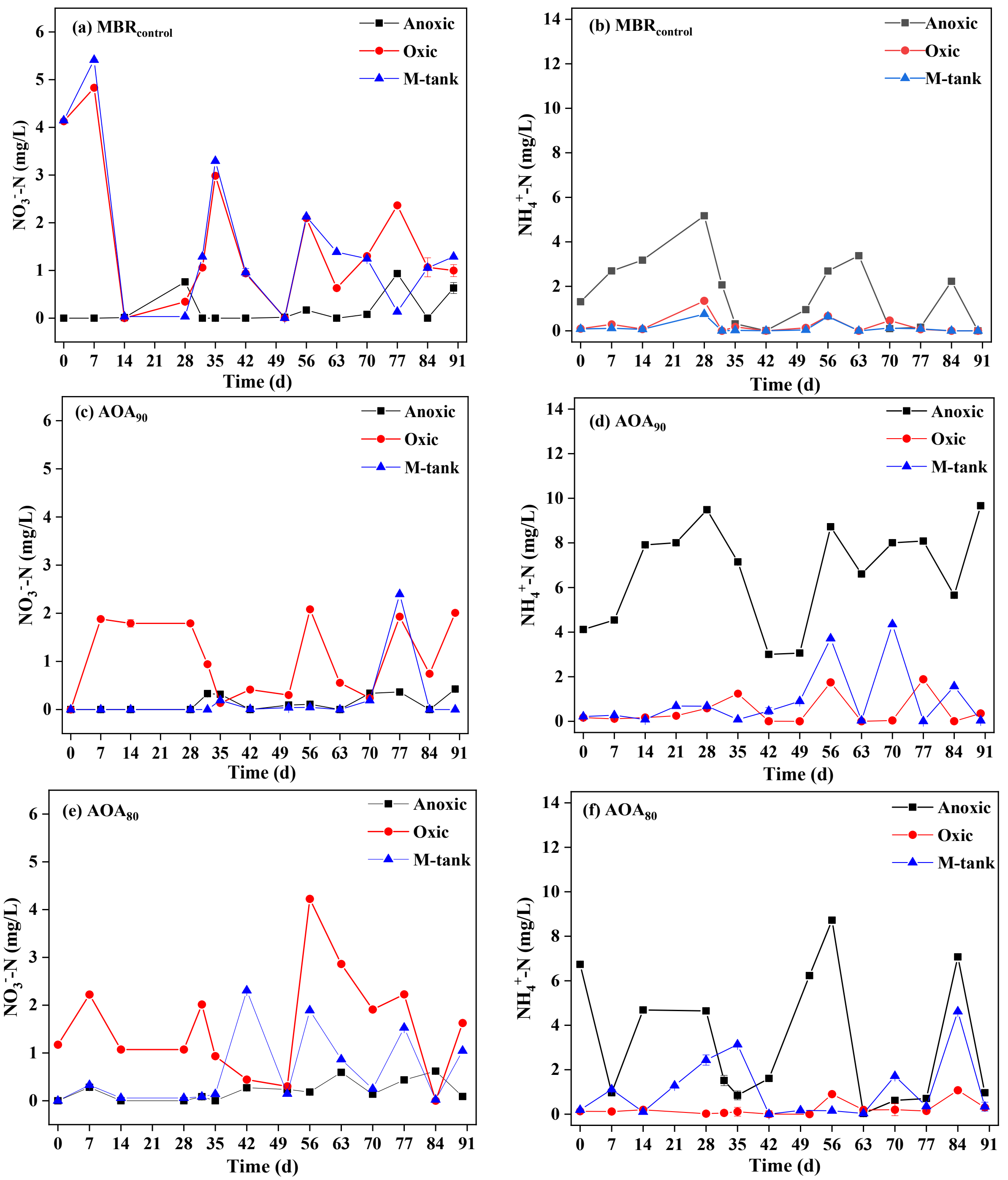

Figure S2. The temporal variations of NO_3^--N and NH_4^+-N in different tanks of $MBR_{control}$, AOA_{90}, and AOA_{80}.

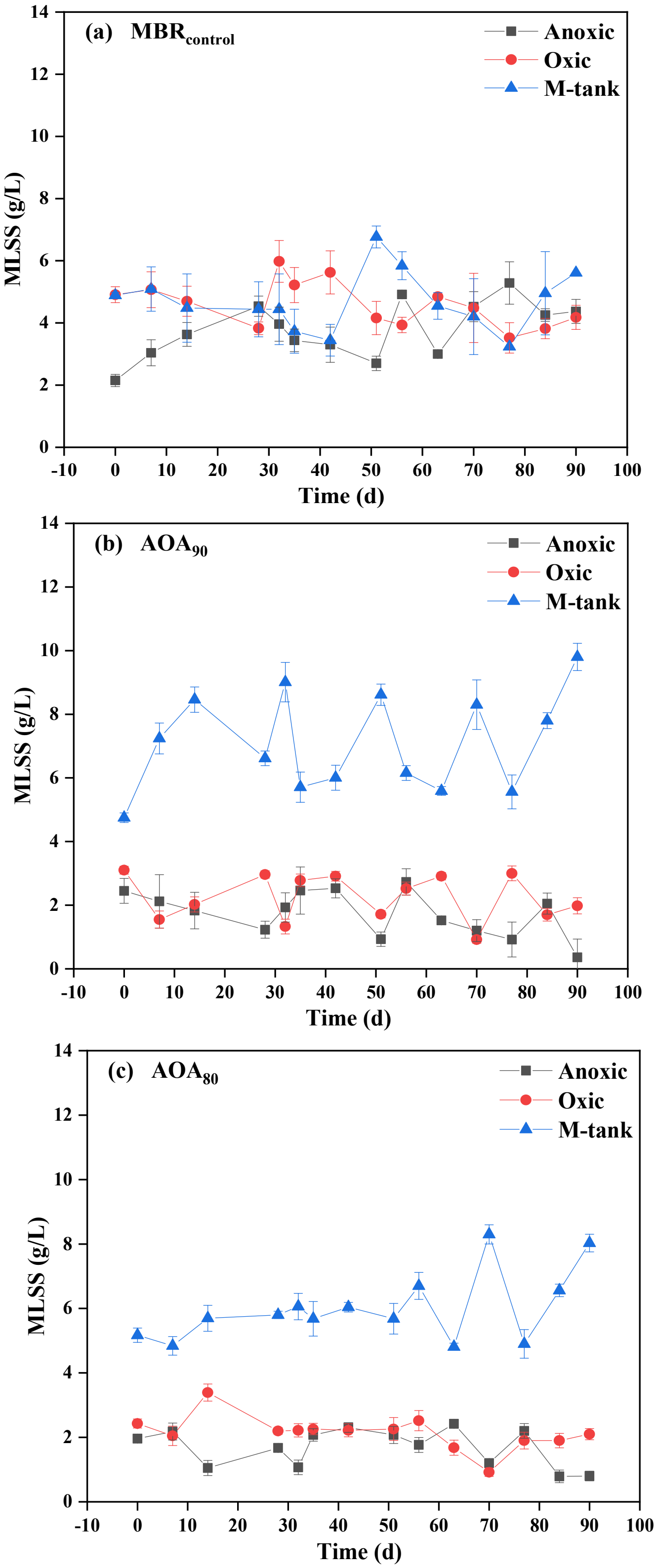

Figure S3. The MLSS concentration in different units of $MBR_{control}$ (a), AOA_{90} (b), and AOA_{80} (c).

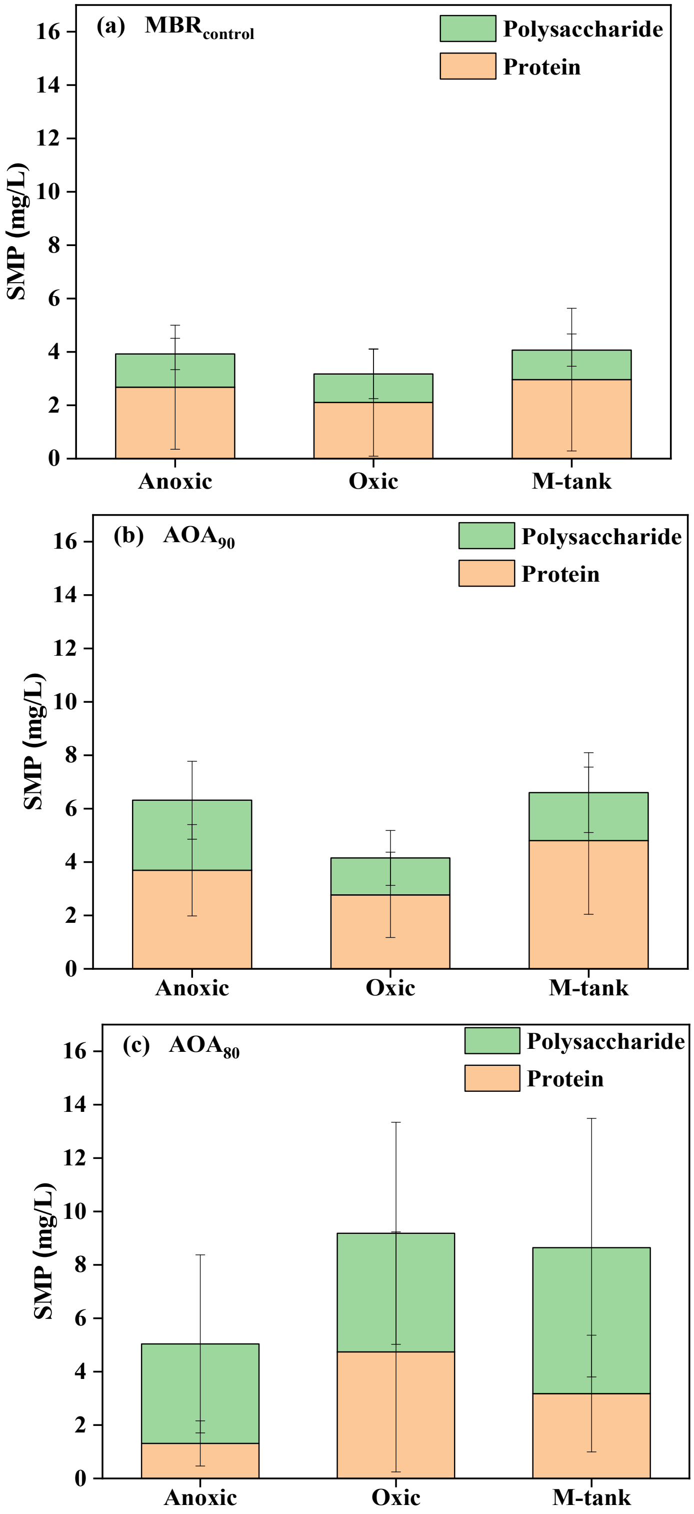

Figure S4. The average polysaccharide and protein in SMP in different units of $MBR_{control}$ (a and b), AOA_{90} (c and d), and AOA_{80} (e and f).

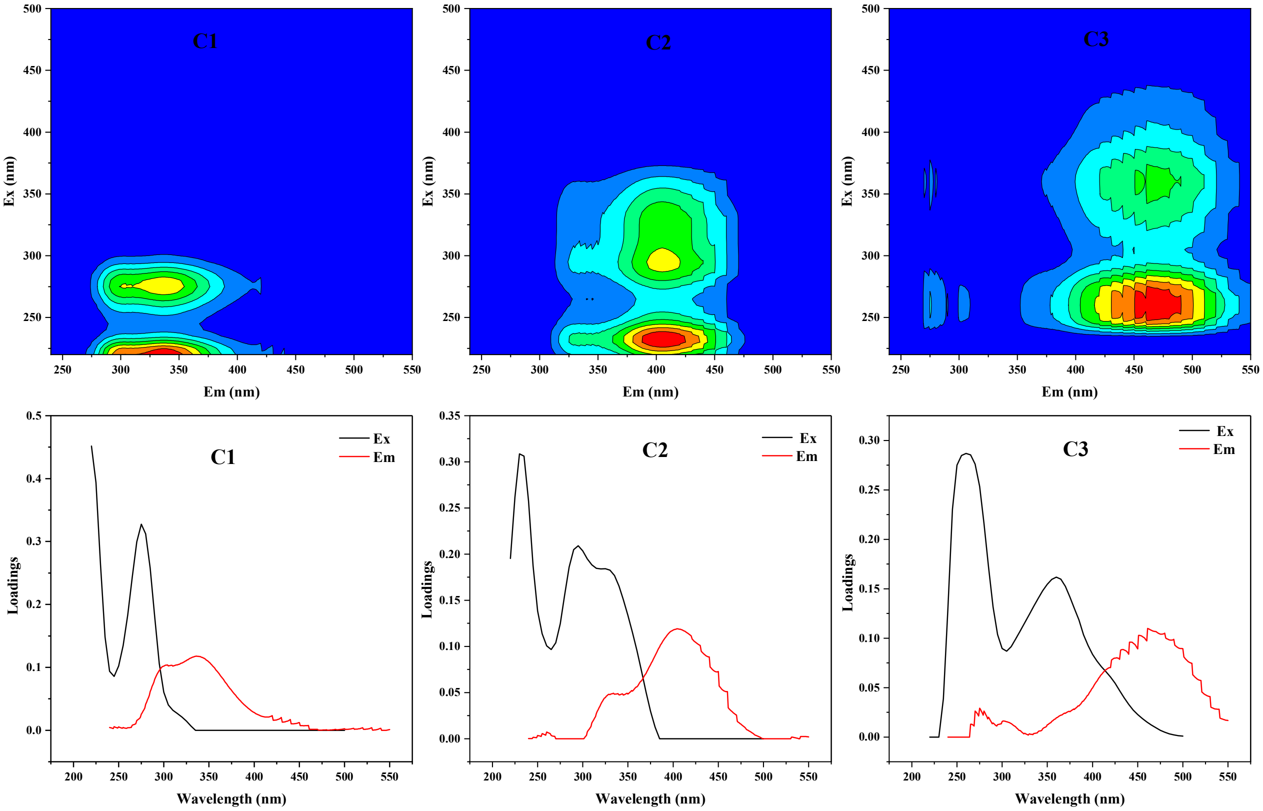

Figure S5. EEM-PARAFAC components, tryptophan-like (C1), fulvic-like (C2), and humic-like (C3).

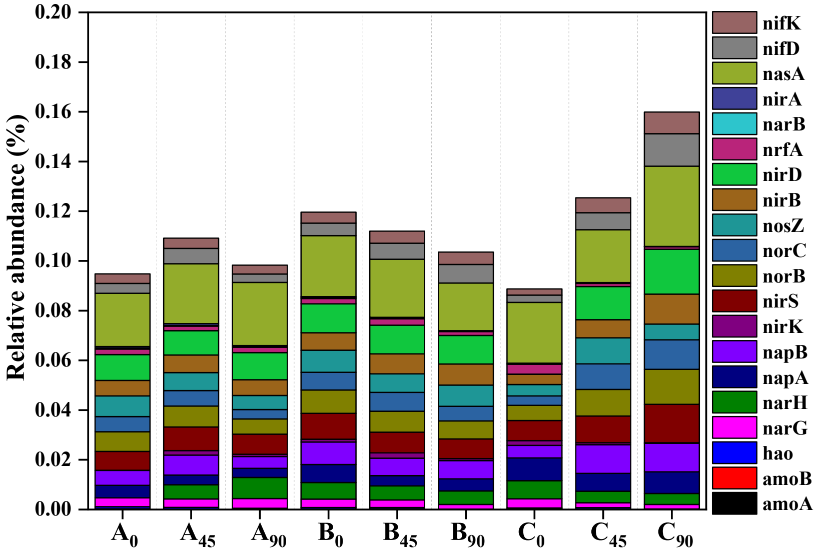

Figure S6. The relative abundance of functional genes responsible for nitrogen metabolism in $MBR_{control}$ (A), AOA_{90} (B), and AOA_{80} (C). (The subscript of A, B, and C means the sampling time.)
